# Reproducibility and Concurrent Validity of Manual Palpation with Rehabilitative Ultrasound Imaging for Assessing Deep Abdominal Muscle Activity: Analysis with Preferential Ratios

**DOI:** 10.3390/diagnostics11020298

**Published:** 2021-02-13

**Authors:** Irene Valentín-Mazarracin, Miriam Nogaledo-Martín, Ibai López-de-Uralde-Villanueva, César Fernández-de-las-Peñas, María Stokes, José L. Arias-Buría, María J. Díaz-Arribas, Gustavo Plaza-Manzano

**Affiliations:** 1 Complete Health of Lawrenceville, Princeton, NJ 08648, USA; vm.irene@hotmail.com; 2 Centro de Fisioterapia Qsana, 28021 Madrid, Spain; miriam.nogaledo@gmail.com; 3 Department of Radiology, Rehabilitation and Physiotherapy, Universidad Complutense de Madrid, 28040 Madrid, Spain; ibailope@ucm.es (I.L.-d.-U.-V.); mjdiazar@med.ucm.es (M.J.D.-A.); gusplaza@ucm.es (G.P.-M.); 4 Department of Physical Therapy, Occupational Therapy, Rehabilitation and Physical Medicine, Universidad Rey Juan Carlos, 28922 Alcorcón, Spain; joseluis.arias@urjc.es; 5 Cátedra Institucional en Docencia, Clínica e Investigación en Fisioterapia: Terapia Manual, Punción Seca y Ejercicio Terapéutico, Universidad Rey Juan Carlos, 28922 Madrid, Spain; 6 School of Health Sciences, University of Southampton, Southampton S017 1BJ, UK; m.stokes@soton.ac.uk; 7 Centre for Sport, Exercise and Osteoarthritis Research Versus Arthritis, Southampton NG7 2UH, UK

**Keywords:** rehabilitative ultrasound imaging, transversus abdominis, reliability, core stability

## Abstract

The abdominal drawing-in maneuver (ADIM) is a clinical tool used for identifying preferential activity of deep abdominal muscles. However, concurrent validity and reproducibility of palpation during the ADIM has not been formally investigated. The aims of this study were (1) to assess intra- and interrater reliability of manual palpation during the ADIM, and (2) to determine the concurrent validity of manual palpation during the ADIM by calculating preferential activation ratio cut-off as assessed with ultrasound imaging (RUSI). Thirty-two subjects (*n* = 16 patients with nonspecific low back pain and 16 comparable healthy individuals) performed the ADIM in a supine hook-lying position. Two experienced assessors evaluated the presence or absence of preferential contraction of the deep abdominal muscles by palpation during the ADIM on 2 different days. Intrarater (test-retest) and interrater reliability of palpation were calculated using Cohen’s kappa coefficients. Muscle thickness of the transverse abdominis (TrA), internal oblique (IO), and external oblique (EO) muscles at rest and during the ADIM were also measured. TrA-Contraction Ratio (TrA-CR), TrA-Preferential Activation Ratio (TrA-PAR), and Modified-TrA-PR (M-TrA-PAR) were calculated. The concurrent validity of manual palpation was determined using the correlation between manual palpation and imaging and by calculating ROC curve (operating characteristics curve), Youden index, and sensitivity and specificity. Intra- and interrater reliability of manual palpation during the ADIM was excellent (*k*: 0.82–1.00) and good to excellent (*k*: 0.71–1.00), respectively. Interrater reliability for muscle thickness ranged from good to excellent (ICC3,1 0.79–0.91). Manual palpation and TrA ratio showed low to moderate correlations (*r*: 0.36–0.60). When evaluating the diagnostic accuracy of manual palpation, the best predictive model (ROC value: 0.89; *p* < 0.001) for correct a preferential contraction of TrA was obtained when the M-TrA-PAR was ≥0.08 (sensitivity: 0.95–1.00; specificity: 0.62). Good to excellent intra- and interrater reliability of manual palpation was found during the ADIM in both patients and healthy groups. Manual palpation showed concurrent validity for identifying the preferential activity of the TrA muscle supporting its use in clinical practice.

## 1. Introduction

Low back pain (LBP) represents a medical problem for the current society, showing a worldwide prevalence ranging from 1.4% to 20% and an incidence from 0.024% to 7% depending on the diagnostic criteria used [1]. In athletes, the 1-year prevalence of LBP can reach 84%, and the lifetime prevalence can reach 94% [2]. In addition, the pooled prevalence rate of health care use of LBP patients is up to 67%, supporting high health care costs [3]. In fact, the estimated annual total cost of LBP in the USA is USD 100 billion [4]. In the Global Burden of Disease Study, LBP was ranked as the fourth leading cause of disability-adjusted life years (DALYs) globally [5,6].

The etiology of LBP is multifactorial. One hypothesis associated with development and recurrence of LBP is a potential dysfunction in the motor control of the muscles of the thoracic cage, e.g., deep abdominal, pelvic floor, multifidus, and diaphragm. Among the abdominal musculature, particular attention has been paid to the deep muscles, e.g., transverse abdominis (TrA) and lumbar multifidi. In fact, training of the TrA and the lumbar multifidi has been advocated to be a relevant component within rehabilitation programs of individuals with LBP, although no linear association between the changes in morphometry or activity of deep abdominal muscles (TrA) and clinical outcomes has been observed [7]. In fact, exercise programs, including training of the deep abdominal muscles, are recommended (moderate quality evidence) for the management of patients with chronic low back pain in clinical practice guidelines [8].

The abdominal drawing-in maneuver (ADIM) is commonly used as a foundational basis of lumbar stabilization training programs. It is hypothesized that the ADIM is able to facilitate coactivity of the deep abdominal musculature and could play a supportive role for the spine during functional activities [9]. In fact, a recent review has concluded that ultrasound imaging and electromyographic activity of the TrA and internal oblique (IO) muscles are highly correlated during the ADIM, supporting the use of this maneuver [10].

Ultrasound (US) imaging is a noninvasive method that allows both the clinician and the patient to observe changes in thickness of the deep abdominal muscles. In fact, the use of rehabilitative ultrasound imaging (RUSI) for visual assessment of deep trunk muscles function, particularly the TrA and lumbar multifidi, and as a biofeedback tool has become increasingly popular [11], particularly in the physical therapy profession [12]. Different systematic reviews have been published about the reliability of RUSI. Hebert et al. concluded that the majority of studies indicate that RUSI exhibits acceptable to good reliability for assessing the TrA. However, they concluded that more high-quality studies are needed [13]. Costa et al. found that current literature reported good to excellent reliability for single measures of TrA thickness but poor to good reliability for measures of thickness changes (reflecting the muscle activity), supporting TrA activity [14].

It should be noted that many clinicians do not have access to US imaging in routine clinical practice and, therefore, manual evaluation remains the most common form of evaluating deep abdominal muscle activity [15]. Before a maneuver can be regularly used in research or clinical practice, its reproducibility and validity must be known. This is particularly relevant for manual assessment during the ADIM, since palpation is operator-dependent. A previous study evaluated interrater reliability of manual palpation during the ADIM in healthy participants, reporting a mean kappa value of 0.52 [16]. The methodology of this small study was poor since no clear analysis was provided. Kaping et al. evaluated the ability of the ADIM for differentiating between people with LBP and healthy participants and found that neither the ADIM nor US imaging was able to discriminate between patients with LBP and healthy participants [17]. No previous study has investigated the validity of manual assessment during the ADIM when compared with a US imaging by determining cut-off values of identifying deep abdominal muscle activity. Therefore, the objectives of the present study were (1) to assess the reliability of manual assessment during the ADIM, and (2) to investigate the concurrent validity of manual assessment by calculating cut-off values assessed with US imaging.

## 2. Materials and Methods

### 2.1. Participants

Consecutive participants with LBP symptoms were recruited via local advertisement and screened for eligible criteria between December 2018 and December 2019. To be included, they had to have a history of nonspecific LBP without lower extremity referral for longer than 1 year. Exclusion criteria included: (1) Current pregnancy; (2) LBP with a specific underlying pathology such as tumor, infection, inflammatory disorders, hernia disc, or prolapsed disc; (3) signs consistent with nerve root compression, e.g., positive straight-leg-raise test <45°, diminished lower-extremity force, or diminished reflexes; (4), lumbar spine surgery; (5) history of osteoporosis; (6) spinal fracture; (7) abdominal pain; and (8) obesity (BMI > 30). The medical history from each patient was solicited from their primary care physician to assess the presence of the exclusion criteria.

In addition, a sample of comparative healthy participants were also recruited. To be eligible to participate, they had to be between 18 and 65 years old and with no history of recurrent LBP and no episode of pain in the previous year. The same exclusion criteria as for the LBP group were applied. The study design was approved by the Review Ethical Committee of Universidad Alcalá de Henares, Spain (CEIM/HU/2017/20). Participants provided written informed consent prior to their inclusion in the study.

Participants were asked (1) to avoid eating or drinking at least 2 h prior to each data collection session, (2) to empty their bladder immediately before each assessment, and (3) to avoid any physical activity the day before the assessment, particularly exercising the abdominal muscles.

### 2.2. Clinical Data

Demographic data, including age, gender, body mass (kg), height (cm), body mass index (BMI), past medical history and location, and nature of symptoms, were collected. Pain intensity and pain-related disability were also assessed using questionnaires. A 100 mm visual analogue scale (VAS, 0: No pain; 100: Maximum pain) was used to assess current intensity of LBP [18]. The Oswestry Disability Index (ODI) was used to determine related disability [19]. The ODI has 10 items referring to activities of daily living that might be disrupted due to LBP. Each item is answered on a 6-point Likert scale ranging from “no problem at all” 0 to “not possible” 5. The total score arises from summing all answers (0–50), multiplied by 2 to obtain a final index ranging from 0 (no disability) to 100 (maximum disability). The Spanish version of the ODI has shown good reliability and internal consistency [20].

### 2.3. Abdominal Drawing-In Maneuver (ADIM)

Participants received a training session where they were taught how to properly perform an abdominal drawing-in maneuver (ADIM). The ADIM involves the drawing-in of the abdominal wall eliciting a concentric contraction of the deep abdominal muscles. It has been claimed that ADIM allows for an efficient and measurable contraction of the TrA [21]. The training session was conducted in quadruped. First, participants were manually assisted in finding their neutral spine position and instructed to perform the ADIM by drawing the umbilicus toward their lumbar spine. This contraction was performed 5 times, holding each contraction for the duration of 2 normal breaths (10 s).

After the training session, participants performed the ADIM twice in the supine hook-lying position as real testing (1) for manual palpation and (2) for ultrasound assessment. The assessor provided the following verbal feedback during the ADIM: “Take a relaxed breath in and out, hold the breath out, and then draw the belly button towards your lumbar spine as you did previously while kneeling on all fours (Supplementary Video).” Participants were advised about avoiding any compensatory pattern, including holding the breath, backward movement of the pelvis, visual costal movement suggestive of superficial abdominal muscles (external oblique, OE) contraction, and pressing the heels down toward the table [22]. Each subject was given a rest period of 2 min between each trial (by different assessors) and a rest period of 10 min between the type of assessments (manual palpation or ultrasound).

### 2.4. Rehabilitative Ultrasound Imaging Assessment

**Figure 1:**
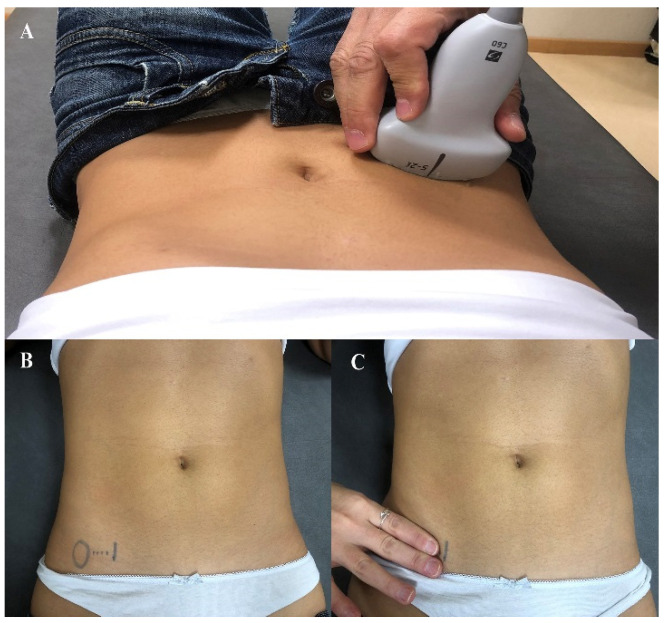
Ultrasound probe placed transversely just above the iliac crest in the midaxillary line (**A**); Manual palpation of the transversus abdominis by placing the index and middle fingers just 2 cm (**B**); medial to the anterior superior iliac spine (**C**).

Ultrasound images were obtained with participants in the supine hook-lying position using a real-time ultrasound scanner (Shenzhen Mindray Co, M7 model, Guangdong Province, China), with a 10-MHz curvilinear probe. For assessing muscle thickness, the probe was placed transversely just above the iliac crest in the midaxillary line (Figure 1A). The focal depth was manually adjusted on each participant, focusing on the OI muscle to maximize optimal visualization of the 3 abdominal muscles (EO, IO, TrA). Three images were obtained bilaterally at rest and on muscle contraction during the ADIM (at the end of exhalation) [23], and the mean of the 3 measurements was used for analysis. For images taken at rest, participants were instructed to breathe normally, and the image was acquired at the end of a regular/normal inspiration to prevent TrA activation [24]. Since all LBP patients exhibited bilateral symptoms, the mean value of both sides was considered in the statistical analysis.

### 2.5. Data Imaging and Management

**Figure 2:**
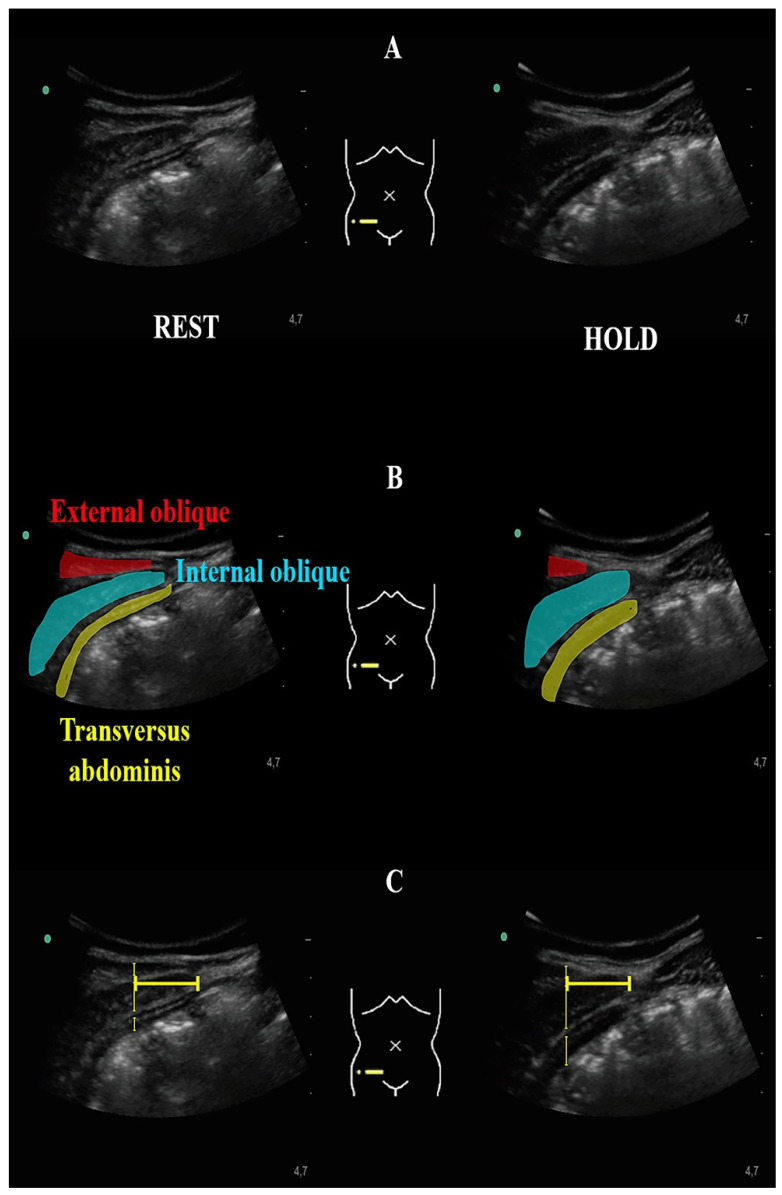
Ultrasound image of the deep abdominal muscles (**A**); Color mark of the deep abdominal muscles (**B**); Ultrasound measurements conducted in a vertical line located 3 cm lateral to the rectus sheath muscle-fascia junction (semilunaris line) (**C**).

Once the image was captured, it was transferred to offline ImageJ 1.8® Software for calculating muscle thickness of the TrA, IO, and EO muscles at rest and during the ADIM (muscle contraction). Muscle thickness was measured as the distance between the inside borders of hyperechoic fascial layers of each muscle separately, without including fascia thickness. To consistently measure over the same point along all the muscles, measurements were conducted in a vertical line located 3 cm lateral to the rectus sheath muscle-fascia junction (semilunaris line) as previously described (Figure 2) [25].

The change in muscle thickness from rest to contraction during the ADIM was reported as an absolute value in millimeters (mm) and as a percentage of change. In addition, the TrA contraction ratio (TrA thickness contracted/TrA thickness at rest) and the TrA preferential activation ratio [(TrA thickness contracted/TrA + OE + OI thickness contracted)−(TrA thickness at rest/TrA + OE + OI thickness at rest)] were also calculated [26]. It has been described that the preferential activation ratio determines the relative coactivation of the TrA in relation to EO and IO muscles. However, since simultaneous activation of the OI and the TrA has been reported [27,28], we created the Modified Preferential Activation Ratio [(TrA thickness contracted/(TrA + OE thickness contracted)−(TrA thickness at rest/TrA + OE thickness at rest)].

### 2.6. Assessors and Reliability Calculation

Two assessors, who received 60 h of education on US imaging and manual palpation, participated. For manual palpation, the assessor placed the index and middle fingers just 2 cm medial to the anterior superior iliac spine, in the anatomical projection of the deep abdominal muscles as is commonly done in clinical practice (Figure 1B). Each assessor determined the presence and/or absence of preferential contraction of the deep abdominal muscles during the ADIM on each subject without knowing the participant’s condition (patient or control). The contraction was considered positive when the assessor perceived a slow, deep, and bilateral tension in the anatomical projection of the deep abdominal muscles, without any compensatory pattern as previously described. Manual palpation was conducted by each assessor on 2 different days with 7 days between. Each day, manual palpation was repeated 3 times by each assessor. A positive contraction was considered when 2 of 3 trials were perceived as having involved contraction. Intrarater reliability was calculated from the assessment of each assessor on both days (test-retest), whereas interrater reliability was calculated from assessment of both assessors on each day.

In addition, ultrasound assessment was conducted once by each assessor to assess interrater reliability of muscle thickness the first day of assessment. Participants were repositioned on each assessment and the order of assessment and raters was numerically randomized. Every image was coded to blind the assessors using alphanumerical codes. We assessed interrater reliability of ultrasound imaging of muscle thickness with the mean data obtained the first day by each assessor.

### 2.7. Sample Size Calculation

A sample of 16 participants per group was calculated for obtaining κ agreement data (proportion of positive ratings: 0.5; Kappa to detect: 0.7; significance level: 0.05; power: 80%; 2-tailed test) as recommended in reliability statistics [29].

### 2.8. Statistical Analysis

Data analysis was performed using SPSS version 21.0 (SPSS Inc., Chicago, IL, USA). For all the analyses, a *p* value < 0.05 was considered as statistically significant. Demographics and clinical characteristics data were assessed for normality by the Shapiro–Wilk test and visual inspection of histograms.

A 2-way mixed-model, consistency-type intraclass correlation coefficient (ICC3,1) was used to evaluate the interrater reliability of muscle thickness (TrA, IO, and EO) measurements on the total sample (as a group and by gender), as well as independently for each group (patients or controls). Based on the ICC2,1 value, reliability was considered excellent (ICC ≥ 0.90), good (0.90 > ICC ≥ 0.70), fair (0.70 > ICC ≥ 0.40), or poor (ICC < 0.40) [30]. The standard error of measurement (SEM) was calculated as follows: SEM (%) = (SDx√1−ICC) × 100. The 90 and 95 minimal detectable change (MDC) were also calculated: MDC90: SEM × 1.64 × √2; MDC95: SEM × 1.96 × √2 [31].

The interrater and test-retest reliability of clinical palpation of the ADIM were evaluated using the Cohen’s kappa (κ) coefficients with their respective 95% Cis, again in the total sample (as a group and by gender) as well as independently for each group (patients or controls). To better understand reliability, observed and expected agreements are also reported along with prevalence and bias indices. Additionally, given that the kappa value is sensitive to imbalances in prevalence and bias, the prevalence-adjusted and bias-adjusted kappa (PABAK) were also calculated [31]. The PABAK calculation is a more restrictive reliability analysis performed by adjusting for high or low prevalence and computing the average of cells a and d in a cross table, substituting this value for the actual values in those cells. Similarly, an adjustment for bias is achieved by substituting the mean of cells b and c for the actual cell values [32]. Reliability was defined according to the classification system proposed by Landis and Koch: Excellent (0.81–1.0), good (0.61–0.80), moderate (0.41–0.60), fair (0.21–0.40), or poor (0.0–0.2) [33].

Concurrent validity was analyzed by evaluating the correlation between the manual palpation with muscle thickness ratios. The correlation was evaluated using Spearman correlation coefficients: Very high correlation (0.9–1.0), high correlation (0.70–0.89), moderate correlation (0.50–0.69), low correlation (0.26–0.49), or small/no-correlation (0.00–0.25) [34]. Furthermore, the validity of the manual palpation during the ADIM to perceive the positive contraction of the deep abdominal muscles was evaluated by comparing the results obtained by palpation by the assessors during the ADIM with muscle thickness ratios. For the analysis, the average of the original and modified preferential activation ratios provided by each assessor was used.

The current gold standard to determine activation of the TrA during the test is fine wire electromyography, which is invasive and was not used. To calculate the optimal cut-off point for TrA preferential activation ratio, we therefore generated a new dichotomous variable based on the relative MDC obtained from the present study data repeated from different days for the total sample. Accordingly, we considered a positive performance of the ADIM if the participant generated a contraction of deep abdominal muscle, i.e., TrA, that exceeded the relative value of the MDC (i.e., error of measurement on repeated testing) established for this muscle at rest but did not exceed the MDC established for the OI and EO muscles. Therefore, by means of a ROC curve (operating characteristics curve) the optimum point for the original and modified preferential activation ratio was established, which can be used as a criterion variable to qualitatively determine the correct performance, as identified by manual palpation, of the ADIM. This cut-off point was determined by means of the Youden index, and the diagnostic precision was evaluated according to the area under the ROC curve, with values above 0.7 being considered acceptable [35]. Finally, using the calculated criterion variable, sensitivity and specificity data of manual palpation of the ADIM applied by each assessor were established. Validity was considered acceptable when at least 70% sensitivity and 50% specificity values were obtained [36].

## 3. Results

### 3.1. Participants

From 25 participants with back pain symptoms responding to the announcement, 9 were excluded due to previous lumbar fusion (*n* = 3), pain radiating to a lower extremity (*n* = 2), pregnancy (*n* = 2), and obesity (*n* = 2), leaving a total of 16 patients. In addition, 16 comparative healthy participants were also included. Demographic and clinical features are shown in Table 1.

### 3.2. Reliability of US Imaging between Assessors

The interrater reliability for muscle thickness ranged from good to excellent for the total sample (ICC ranging from 0.84 to 0.90), without significant differences between males (ICC ranging from 0.77 to 0.90) and females (ICC ranging from 0.78 to 0.90) or between patients (ICC from 0.79 to 0.90) and healthy participants (ICC from 0.86 to 0.91). In general, interrater reliability was slightly higher in the healthy group than in the LBP patient group. The TrA exhibited the lowest SEM and MDC (SEM: 0.36–0.75; MDC90–95: 0.84–2.08), followed by the EO (SEM: 0.36–0.88; MDC90–95: 0.84–2.44) and IO (SEM: 0.61–1.66; MDC90–95: 1.42–3.22). Again, no significant gender differences were found. In general, the SEM and MDC for muscle thickness during the ADIM were higher than at rest and also slightly higher in patients than in controls. The descriptive statistics, ICCs, and associated 95% confidence intervals, SEM, MDC90, and MDC95, are shown in Table 2.

### 3.3. Reliability of Manual Palpation during the ADIM

The interrater reliability of the ADIM ranged from good to excellent regardless of the total sample, gender, or group (*k*: 0.71–1.00), while the test-retest (intrarater) reliability was excellent (*k*: 0.82–1.00). Interrater reliability was good (*k*: 0.71) in patients with LBP, while it was almost perfect (*k*: 0.82–1.00) in controls. Importantly, reliability was not affected by the prevalence and sample bias indices since PABAK and kappa index values were practically identical (Table 3).

### 3.4. Concurrent Validity of Manual Palpation

Transformation of muscle thickness to activation ratios showed that the Transversus Abdominis-Contraction Ratio (TrA-CR) was 1.88 (0.49), the TrA-Preferential Activation Ratio (TrA-PAR) was 0.08 (0.05), and the Modified-TrA-Preferential Activation Ratio (M-TrA-PAR) was 0.09 (0.1).

Manual palpation during the ADIM showed acceptable concurrent validity as it showed low to moderate correlations with ratios of the US imaging measurement (Table 4). In fact, manual evaluation during the ADIM showed the highest association with the M-TrA-PAR (Table 4).

Concurrent validity data from manual palpation during the ADIM can be found in Table 5. The transformation of continuous to dichotomous US imaging measurement to determine the selective contraction of TrA showed significant and acceptable models for the preferential activation indices (TrA-PAR (ROC value = 0.86; *p* = 0.001) and M-TrA-PAR (ROC value = 0.89; *p* < 0.001). When evaluating the diagnostic accuracy of manual palpation, the best result was obtained when a value equal to or greater than 0.08 was observed in the M-TrA PAR index and used as a criterion variable for correct selective contraction of TrA (sensitivity: 0.95–1.00; specificity: 0.62).

## 4. Discussion

The present results revealed a good to excellent intra- and interrater reliability of palpation and interrater reliability for muscle thickness assessment during the ADIM in patients with LBP and healthy controls. Manual palpation showed concurrent validity for identifying the preferential activity of the deep abdominal muscles, potentially the TrA and IO, supporting its use in clinical practice.

### 4.1. Reliability of Manual Palpation during ADIM and RUSI Imaging

This is the first study investigating both intra- and interrater reliability of manual palpation during ADIM. We found excellent intrarater reliability on different days (κ: 0.82–1.00) and good to excellent interrater reliability (*k*: 0.71–1.00). The current results are better than those previously reported by an earlier study evaluating the interrater reliability of manual palpation during the ADIM in healthy subjects (*k*: 0.52) [16]. The previous study included students, and the statistical analysis only included single kappa coefficients [16]. More recently, Kaping et al. investigated the interrater reliability of manual palpation during the ADIM and reported substantial agreement (*k*: 0.71, 95% CI 0.41 to 1.00) [17]. Current and previous findings would suggest that manual palpation during the ADIM seems to be a reliable procedure for identifying deep abdominal muscles activity. In addition, an important topic of the present analysis is that reliability values were not affected when the PABAK test, a more restrictive reliability statistical analysis, was applied. The application of PABAK has been shown to increase the reliability of clinical tests for identifying shoulder instability [37].

This is the first study using PABAK calculations to determine the reliability of manual palpation during the ADIM, which increased the precision of statistical analysis. Nevertheless, we should consider that both assessors of our study received 60 h of education on RUSI and manual palpation during the ADIM, which could explain their positive results. This is a relevant topic since manual palpation and RUSI assessment are operator-dependent. Future studies should investigate the role of proper education and clinical experience in reproductivity of manual palpation during the ADIM to determine if reliability is related to the assessor experience.

We also found good to excellent interrater reliability of RUSI assessment of the deep abdominal muscles in both LBP patients (ICC 0.79–0.90) and healthy participants (ICC 0.86–0.91). Two systematic reviews have previously investigated reliability of RUSI of the abdominal muscles [13,14]. Hebert et al. concluded that most studies investigating intrarater reliability showed moderate to excellent score (ICC from 0.48 to 1.0). However, 60% of the studies were of poor methodological quality [13]. Interrater reliability was investigated in only two studies, which reported excellent scores (ICC from 0.91 to 1.0). However, data were mainly based on healthy populations [13]. Similar results were reported by the review by Costa et al. [14]. Costa et al. observed good to excellent reliability (ICC 0.81 to 0.92) for single measures of TrA thickness but poor to good reliability (ICC 0.48 to 0.78) for measures of thickness changes, reflecting TrA muscle activity [14]. The present results, showing good to excellent interrater reliability of muscle thickness measurements, agree with these previous reviews [13,14]. Importantly, our study also observed that interrater reliability was good to excellent in LBP (ICC 0.79 to 0.90).

### 4.2. Concurrent Validity of Manual Palpation Compared with RUSI Imaging

One of the main objectives of this study was to determine the concurrent validity of palpation during the ADIM for evaluating deep abdominal muscle activity. We found low to moderate association between manual palpation with the different US ratios (see Table 4). We used three different preferential activation US ratios (TrA-CR, TrA-PAR, M-TrA-PAR) to assess the concurrent validity of manual palpation. The highest association between palpation and US ratios was observed for the M-TrA-PAR (*r*: 0.60), whereas the lowest association was reported for the TrA-CR (*r*: 0.36). In fact, the application of the Youden index identified that TrA-CR was not a significant predictor of TrA contraction perceived during the ADIM by the assessor. On the contrary, both preferential activation ratios (TrA-PAR, M-TrA-PAR) showed acceptable models. These present results support that preferential activation ratios, instead the TrA contraction ratio, should be used in clinical practice for evaluating deep abdominal muscle activity. These results agree with those previously reported by Pulkovski et al., who also found that the TrA-CR was not able to distinguish between patients with LBP and controls (discriminant validity) [38].

Interestingly, although both preferential activation ratios (TrA-PAR and M-TrA-PAR) showed concurrent validity with manual palpation, the best predictive model was obtained with the M-TrA-PAR. This modified preferential activation ratio considers the simultaneous contraction of the TrA and IO muscles [26,27], which could explain its better concurrent validity with manual palpation. One potential explanation may be the anatomical association between the TrA and IO muscles and, therefore, the inability to directly palpate the TrA [39]. Future studies should investigate the discriminant validity of this preferential activation ratio (M-TrA-PAR) for identifying patients with LBP from healthy controls.

### 4.3. Strengths and Limitations

The current results should be considered attending to the strengths and limitations of the study. A strength of the present study was its inclusion of most of the methodological flaws identified by Costa et al. [14]. Costa et al. identified that inappropriate description of the sample and the assessors, inadequate statistics, and a lack of blinding or lack of controlling measures order were the most common methodological weaknesses of published studies [14]. The current study was conducted considering these weaknesses to improve the methodological quality. Additionally, we conducted a deep analysis of reproducibility and concurrent validity of manual palpation of deep abdominal muscles activity during the ADIM as compared with RUSI measures. Although we followed previous guidelines for reliability studies [28], the total sample size could be considered relatively small. Naturally, this could affect the presence of positive and negative findings up to 50%. However, to overcome this situation, PABAK calculations were also included and reported along with kappa to transparently show how data would have been with equal distributions of positive and negative test results.

Among the potential limitations, manual palpation and RUSI measurements were not conducted simultaneously. This situation could affect the results considering a possible learning effect on the participants. The topic of manual palpation of the deep abdominal muscles is currently debatable, since it is not possible to directly palpate the TrA muscle due to its deep anatomical location. Therefore, it should be comprehensible that manual palpation during the ADIM aims to identify preferential activity of the deep abdominal muscles, that is, TrA and IO, against activity of the most superficial muscle, i.e., the EO muscle. Additionally, it should be noted that MCD and SEM data from muscle thickness data used in this study to calculate the optimal cut-off point for TrA preferential activation ratio were based on interrater, and not intrarater, reliability. The reason for not assessing intrarater reliability of US imaging muscle thickness is the literature has well established it as being good to excellent [13,14]. Finally, the rigid inclusion criteria used in the study may limit the generalizability of the results. Additionally, data by gender in the current study should be considered with caution at this stage, since it was not possible to determine reliability of the data by gender in each group (patients or controls) due to the sample size.

## 5. Conclusions

The current study reported good to excellent intra- and interrater reliability of manual palpation and interrater reliability of muscle thickness assessment during the ADIM in patients with LBP and healthy controls. Manual palpation during the ADIM showed concurrent validity with RUSI measures for identifying preferential activity of the deep abdominal muscles, supporting the use of palpation in clinical practice. Future studies should investigate if the inclusion of preferential activation ratios provides better information of changes during the course of treatment in patients with LBP.

## Figures and Tables

**Table 1:** Demographic and clinical characteristics of the sample.

Outcome	Asymptomatic Controls (*n* = 16)	Low Back Pain (*n* = 16)	*p*-Value
	Mean ± SD/*n* (%)	Mean ± SD/*n* (%)	
Age (years)	29.5 ± 4.5	28.5 ± 4.0	0.648
Gender (female)	8 (50%)	8 (50%)	1.00
Body mass (kg)	68.0 ± 9.5	67.0 ± 13.0	0.854
Height (m)	1.75 ± 0.1	1.70 ± 0.1	0.266
BMI (kg/m^2^)	22.3 ± 2.0	22.9 ± 2.4	0.418
VAS (0–100, mm)	–	39.5 ± 15.9	–
ODI (0–100, %)	–	24.5 ± 14.5	–

BMI: Body Mass Index; VAS: Visual Analog Scale; ODI: Oswestry Disability Index.

**Table 2:** Interrater reliability of ultrasound muscle thickness at rest and during the ADIM.

Transversus Abdominis (TrA)						
Outcomes	Mean ± SD		ICC (95 CI)	SEM (mm)	MDC90 (mm; %)	MDC95 (mm; %)
	Rater A	Rater B				
Total Sample (*n* = 32)						
Rest (mm) ADIM (mm) Change (mm; %)	3.5 ± 0.8 6.2 ± 1.4 2.7 ± 1.4; 77%	3.4 ± 0.8 6.2 ± 1.5 2.8 ± 1.3; 84%	0.84 (0.67 to 0.92) 0.86 (0.71 to 0.93)	0.43 0.72	1.01; 29% 1.68; 27%	1.20; 35% 1.99; 32%
Male Subjects (*n* = 16)						
Rest (mm) ADIM (mm) Change (mm; %)	3.9 ± 0.6 6.4 ± 1.3 2.5 ± 1.5; 64%	3.8 ± 0.5 6.5 ± 1.4 2.7 ± 1.3; 71%	0.77 (0.36 to 0.92) 0.89 (0.69 to 0.96)	0.33 0.61	0.76; 20% 1.42; 22%	0.90; 23% 1.69; 26%
Female Subjects (*n* = 16)						
Rest (mm) ADIM (mm) Change (mm; %)	3.1 ± 0.9 5.9 ± 1.5 2.8 ± 1.3; 90%	3.0 ± 0.9 5.9 ± 1.5 2.9 ± 1.4; 97%	0.78 (0.36 to 0.92) 0.83 (0.50 to 0.94)	0.54 0.83	1.26; 41% 1.95; 33%	1.50; 49% 2.31; 39%
Asymptomatic Subjects (*n* = 16)						
Rest (mm) ADIM (mm) Change (mm; %)	3.6 ± 0.8 6.3 ± 1.6 2.7 ± 1.5; 77%	3.4 ± 0.8 6.3 ± 1.6 2.8 ± 1.5; 83%	0.89 (0.68 to 0.96) 0.89 (0.68 to 0.96)	0.360.75	0.84; 24% 1.75; 28%	1.00; 29% 2.08; 33%
Low Back Pain Patients (*n* = 16)						
Rest (mm) ADIM (mm) Change (mm; %)	3.4 ± 0.8 6.0 ± 1.1 2.6 ± 1.3; 76%	3.3 ± 0.9 6.12± 1.3 2.8 ± 1.2; 85%	0.79 (0.40 to 0.93) 0.81 (0.47 to 0.94)	0.51 0.070	1.19; 35% 1.64; 27%	1.41; 42% 1.95; 32%
Internal Oblique (IO)						
Total Sample (*n* = 32)						
Rest (mm) ADIM (mm) Change (mm; %)	6.6 ± 1.3 8.0 ± 2.1 1.5 ± 2.0; 22%	6.6 ± 1.5 8.3 ± 2.2 1.7 ± 1.9; 26%	0.90 (0.80 to 0.95) 0.85 (0.69 to 0.93)	0.62 1.10	1.44; 22% 2.58; 32%	1.71; 26% 3.06; 37%
Male Subjects (*n* = 16)						
Rest (mm) ADIM (mm) Change (mm; %)	7.1 ± 1.0 8.2 ± 2.3 1.2 ± 1.4; 15%	7.1 ± 1.3 8.5 ± 1.9 1.2 ± 1.1; 20%	0.86 (0.60 to 0.95) 0.83 (0.52 to 0.94)	0.57 1.15	1.34; 19% 2.67; 32%	1.59;22% 3.17; 38%
Female Subjects (*n* = 16)						
Rest (mm) ADIM (mm) Change (mm; %)	6.0 ± 1.4 7.9 ± 2.1 1.9 ± 1.3; 32%	6.0± 1.6 8.1 ± 2.4 2.1 ± 1.9; 35%	0.90 (0.70 to 0.96) 0.87 (0.62 to 0.95)	0.68 1.10	1.58; 26% 2.56; 32%	1.87; 31% 3.04; 38%
Asymptomatic Subjects (*n* = 16)						
Rest (mm) ADIM (mm) Change (mm; %)	7.0 ± 1.3 8.5 ± 2.2 1.5 ± 2.3; 22%	7.1 ± 1.5 8.6 ± 2.1 1.5 ± 2.1; 22%	0.90 (0.70 to 0.96) 0.86 (0.60 to 0.95)	0.64 1.07	1.49; 21% 2.50; 29%	1.77;25% 2.97; 35%
Low Back Pain Patients (*n* = 16)						
Rest (mm) ADIM (mm) Change (mm; %)	6.2 ± 1.3 7.6 ± 2.0 1.4 ± 1.7; 23%	6.1 ± 1.4 7.9 ± 2.3 1.9 ± 1.8; 31%	0.89 (0.68 to 0.96) 0.83 (0.52 to 0.94)	0.61 1.16	1.42; 23% 2.71; 35%	1.68; 27% 3.22; 41%
External Oblique (EO)						
Total Sample (*n* = 32)						
Rest (mm) ADIM (mm) Change (mm; %)	4.7 ± 1.1 5.6 ± 1.5 0.9 ± 1.4; 19%	4.6 ± 1.2 5.6 ± 1.6 1.0 ± 1.5; 23%	0.90 (0.80 to 0.95) 0.84 (0.68 to 0.92)	0.45 0.83	1.06; 23% 1.94; 35%	1.26; 27% 2.31; 41%
Male Subjects (*n* = 16)						
Rest (mm) ADIM (mm) Change (mm; %)	5.0 ± 1.0 5.6 ± 1.4 0.6 ± 1.4; 12%	4.8 ± 1.2 5.7 ± 1.8 0.9 ± 1.8; 19%	0.90 (0.72 to 0.96) 0.89 (0.68 to 0.96)	0.47 0.73	1.09; 22% 1.70; 30%	1.30; 26% 2.02; 36%
Female Subjects (*n* = 16)						
Rest (mm) ADIM (mm) Change (mm; %)	4.4 ± 1.1 5.6 ± 1.7 1.2 ± 1.4; 27%	4.3 ± 0.9 5.5 ± 1.6 1.2 ± 1.1; 28%	0.89 (0.69 to 0.96) 0.81 (0.44 to 0.93)	0.45 0.94	1.05; 24% 2.20; 40%	1.24; 29% 2.61; 47%
Asymptomatic Subjects (*n* = 16)						
Rest (mm) ADIM (mm) Change (mm; %)	4.9 ± 1.0 5.9 ± 1.6 1.0 ± 1.6; 20%	4.6 ± 1.0 5.8 ± 1.8 1.2 ± 1.6; 25%	0.91 (0.72 to 0.97) 0.88 (0.65 to 0.96)	0.36 0.80	0.84; 18% 1.87; 32%	1.00; 21% 2.22; 38%
Low Back Pain Patients (*n* = 16)						
Rest (mm) ADIM (mm) Change (mm; %)	4.5 ± 1.2 5.2 ± 1.4 0.8 ± 1.2; 17%	4.5 ± 1.2 5.4 ± 1.5 0.9 ± 1.4;20%	0.90 (0.71 to 0.97) 0.79 (0.41 to 0.93)	0.52 0.88	1.21; 27% 2.05; 38%	1.44; 32% 2.44;46%

ADIM: Abdominal Drawing-In Maneuver; CI: Confidence Interval; ICC: Intraclass Correlation Coefficient; MDC: Minimal Detectable Change; SEM: Standard Error of the Measurement.

**Table 3:** Interrater and intrarater test-retest reliability of manual palpation during the abdominal drawing-in maneuver (ADIM).

Interrater Reliability						
Outcome	Observed Agreement	Expected Agreement	Prevalence Index	Bias Index	Kappa (95% CI)	PABAK
Day 1						
Total Sample (*n* = 32)	0.91	0.61	0.47	0.03	0.76 (0.50 to 1.00)	0.81
Males (*n* = 16)	0.94	0.59	0.44	0.06	0.85 (0.55 to 1.00)	0.88
Females (*n* = 16)	0.88	0.63	0.50	0.01	0.67 (0.23 to 1.00)	0.75
Asymptomatic (*n* = 16)	0.94	0.66	0.56	0.06	0.82 (0.47 to 1.00)	0.88
Low Back Pain (*n* = 16)	0.88	0.56	0.38	0.13	0.71 (0.34 to 1.00)	0.75
Day 2						
Total Sample (*n* = 32)	0.94	0.63	0.50	0.00	0.83 (0.61 to 1.00)	0.88
Males (*n* = 16)	0.94	0.59	0.44	0.06	0.85 (0.55 to 1.00)	0.88
Females (*n* = 16)	0.94	0.66	0.56	0.06	0.82 (0.47 to 1.00)	0.88
Asymptomatic (*n* = 16)	1.00	0.70	0.63	0.00	1.00 (1.00 to 1.00)	1.00
Low Back Pain (*n* = 16)	0.88	0.57	0.38	0.00	0.71 (0.33 to 1.00)	0.75
**Intrarater Test-Retest Reliability**						
**Outcome**	**Observed Agreement**	**Expected Agreement**	**Prevalence Index**	**Bias Index**	**Kappa (95% CI)**	**PABAK**
Rater A						
Total Sample (*n* = 32)	0.94	0.63	0.5	0	0.83 (0.61 to 1.00)	0.88
Males (*n* = 16)	0.94	0.59	0.44	0.06	0.85 (0.55 to 1.00)	0.88
Females (*n* = 16)	0.94	0.66	0.56	0.06	0.82 (0.47 to 1.00)	0.88
Asymptomatic (*n* = 16)	0.94	0.66	0.56	0.06	0.82 (0.47 to 1.00)	0.88
Low Back Pain (*n* = 16)	0.94	0.59	0.44	0.06	0.85 (0.55 to 1.00)	0.88
Rater B						
Total Sample (*n* = 32)	0.97	0.61	0.47	0.03	0.92 (0.77 to 1.00)	0.94
Males (*n* = 16)	0.94	0.59	0.44	0.06	0.85 (0.55 to 1.00)	0.88
Females (*n* = 16)	1	0.63	0.5	0	1.00 (1.00 to 1.00)	1
Asymptomatic (*n* = 16)	1	0.7	0.63	0	1.00 (1.00 to 1.00)	1. 00
Low Back Pain (*n* = 16)	0.94	0.55	0.31	0.06	0.86 (0.60 to 1.00)	0.88

CI: Confidence Interval; PABAK: Prevalence-Adjusted and Bias-Adjusted Kappa.

**Table 4:** Correlations between manual palpation and muscle thickness assessing Transversus Abdominis (TrA) Muscle Contraction.

Rater	TrA-CR	TrA-PAR	M-TrA-PAR
Rater A	0.39 *	0.48 *	0.60 **
Rater B	0.36 *	0.39 *	0.51 **

TrA-CR: Transversus Abdominis-Contraction Ratio; TrA-PAR: Transversus Abdominis-Preferential Activation Ratio; M-TrA-PAR: Modified-Transversus Abdominis-Preferential Activation Ratio. * *p* < 0.05. ** *p* < 0.01.

**Table 5:** Concurrent validity of manual palpation to determine the contraction of the Transversus Abdominis using USI measurements transformed into dichotomous ones as a criterion variable.

**TrA-CR**				
Proposed model for the dichotomous transformation of USI measurements: ROC value (95% CI) = 0.60 (0.39–0.82); *p* = 0.33 Youden Index = 0.25 TrA-CR Cut-off point = 1.32				
TrA-PAR *				
Proposed model for the dichotomous transformation of USI measurements: ROC value (95% CI) = 0.86 (0.71–1.00); *p* = 0.001 Youden Index = 0.73 TrA-PAR Cut-off point = 0.06				
**Rater**	Sensitivity (95% CI)	Specificity (95% CI)	LR + (95% CI)	LR-(95% CI)
ADIM				
Rater A	0.90 (0.77–1.00)	0.33 (0.12–0.55)	1.35 (1.00–1.93)	0.30 (0.07–1.00)
Rater B	0.85 (0.69–1.00)	0.50 (0.22–0.78)	1.70 (1.00–3.08)	0.30 (0.09–0.98)
M-TrA-PAR *				
Proposed model for the dichotomous transformation of USI measurements: ROC value (95% CI) = 0.89 (0.74–1.00); *p* < 0.001 Youden Index = 0.78 M-TrA-PAR Cut-off point = 0.08				
**Rater**	Sensitivity (95% CI)	Specificity (95% CI)	LR + (95% CI)	LR-(95% CI)
ADIM				
Rater A	1.00 (1.00–1.00)	0.62 (0.35–0.88)	2.60 (1.31–5.17)	0.00 (0.00–0.00)
Rater B	0.95 (0.85–1.00)	0.62 (0.35–0.88)	2.46 (1.23–4.94)	0.09 (0.01–0.60)

ADIM: Abdominal Drawing-In Maneuver; TrA-CR: Transversus Abdominis-Contraction Ratio; TrA-PAR: Transversus Abdominis-Preferential Activation Ratio; M-TrA-PAR: Modified-Transversus Abdominis-Preferential Activation Ratio. * Model statistically significant.

## Supplementary Materials

The following are available online at https://www.mdpi.com/2075-4418/11/2/298/s1.
